# Supplementary material for: The Impact of High BMI on Pregnancy Outcomes and Complications in Women with PCOS Undergoing IVF—A Systematic Review and Meta-Analysis
Source: J Clin Med. 2024 Mar 10;13(6):1578. doi: 10.3390/jcm13061578 (PMC10970739; doi:10.3390/jcm13061578)
Supplement: Supplementary file 1 [file jcm-13-01578-s001.zip › jcm-2902292-Supplementary.pdf]

Supplementary:

Table S1. The Newcastle-Ottawa Scale (NOS) quality assessment of the included studies

1ST Author, Year	Selection of cohorts ****				Comparability of cohorts **	Outcome ***			NOS Score
	Representativeness of the exposed cohort (with high BMI)	Selection of the non-exposed cohort (without high BMI)	Ascertainment of exposure	Demonstration that outcome of interest was not present at start of study	Comparability of cohorts on the basis of the design or analysis	Assessment of outcome	Was follow up long enough for outcomes to occur	Adequacy of follow up of cohorts	
**McCormick, 2008 [18]**	*	*	*	*	*	*	*	*	8
**Ozgun, 2011 [21]**	*	*	*	*	*	*	*	*	8
**Huang, 2014 [17]**	*	*	*	*		*	*	*	7
**Bailey, 2014 [23]**	*	*	*	*	*	*	*	*	8
**Cui, 2016 [24]**	*	*	*	*		*	*		6
**Sheng, 2017 [27]**	*	*	*	*		*	*	*	7
**Pan, 2018 [29]**	*	*	*	*		*	*		6
**Yang, 2018 [31]**	*	*	*	*		*	*	*	7
**Chen, 2018 [28]**	*	*	*	*	*	*	*		7
**Qiu, 2019 [15]**	*	*	*	*		*	*		6
**Zhou, 2020 [14]**	*	*	*	*	*	*	*		7
**Rehman, 2018 [30]**	*	*	*	*	*	*	*	*	
**Kalem, 2016 [25]**	*	*	*	*		*	*	*	7
**Bu, 2013 [22]**	*	*	*	*		*	*	*	7
**Ae Ra Han, 2011 [16]**	*	*	*	*	*	*	*	*	8
**Kamardi, 2021 [19]**	*	*	*	*	*	*	*	*	8
**Abdulkhalikova, 2020 [33]**	*	*	*	*		*	*		6
**Liu, 2020 [32]**	*	*	*	*		*	*	*	7
**TRENKIĆ, 2016 [26]**	*	*	*	*	*	*	*	*	8

The effect of Pre-Pregnancy BMI on Multi-pregnancy among PCOS women:

Pooled data from four studies (n=1277) found no statistical difference between PCOS women with high BMI undergoing IVF compared with those with normal BMI (OR 1.12 [95% CI 0.88–1.43]; z = 0.95; p = 0.34; I^2 = 0%). No observed heterogeneity among the included studies.

	Normal BMI		High BMI			Odds Ratio	
**Study or Subgroup**	**Events**	**Total**	**Events**	**Total**	**Weight**	**M-H, Fixed, 95% CI**	**Year**
Ae Ra Han 2011	82	272	18	64	16.3%	1.10 [0.60, 2.02]	2011
Kalem 2016	48	90	49	110	16.4%	1.42 [0.81, 2.49]	2016
Yang 2018	48	117	28	67	16.8%	0.97 [0.53, 1.78]	2018
Zhou 2020	100	225	141	332	50.5%	1.08 [0.77, 1.52]	2020
**Total (95% CI)**		**704**		**573**	**100.0%**	**1.12 [0.88, 1.43]**	
Total events	278		236				
Heterogeneity: Chi² = 0.96, df = 3 (P = 0.81); I² = 0%							
Test for overall effect: Z = 0.95 (P = 0.34)							

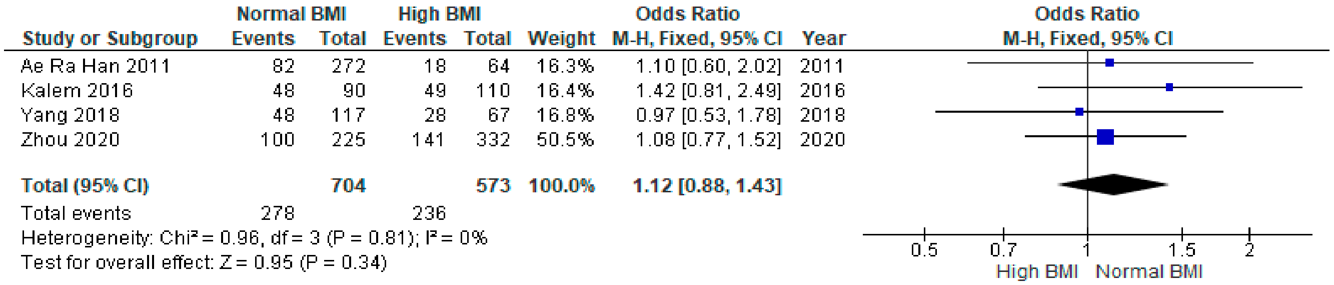

Figure S1. Multiple pregnancy rates pooled analysis of 4 studies.
